# lncRNA TPT1-AS1 promotes cell migration and invasion in esophageal squamous-cell carcinomas by regulating the miR-26a/HMGA1 axis

**DOI:** 10.1515/med-2022-0533

**Published:** 2023-02-14

**Authors:** Wenhua Cheng, Fang Yang, Yong Ma

**Affiliations:** The 3rd Department of Digestion, Shanxi Province Cancer Hospital, Shanxi Hospital Affifiliated to Cancer Hospital, Chinese Academy of Medical Sciences, Cancer Hospital Affifiliated to Shanxi Medical University, Taiyuan City, Shanxi Province, 030013, P. R. China; Radiotherapy Head and Neck Department, Shanxi Province Cancer Hospital, Shanxi Hospital Affifiliated to Cancer Hospital, Chinese Academy of Medical Sciences, Cancer Hospital Affifiliated to Shanxi Medical University, Taiyuan City, Shanxi Province, 030013, P. R. China; The 2nd Department of Chest Surgery, Shanxi Province Cancer Hospital, Shanxi Hospital Affifiliated to Cancer Hospital, Chinese Academy of Medical Sciences, Cancer Hospital Affifiliated to Shanxi Medical University, No. 3 Workers Xin Jie, Xinghualing District, Taiyuan City, Shanxi Province, 030013, P. R. China

**Keywords:** esophageal squamous-cell carcinomas, lncRNA TPT1-AS1, miR-26a, HMGA1, migration, invasion

## Abstract

lncRNA TPT1-AS1 plays an oncogenic role in ovarian and cervical cancers. However, its involvement in the pathological progress of esophageal squamous-cell carcinomas (ESCCs) is unclear. lncRNA TPT1-AS1 was mainly localized in the cytoplasm of ESCC cells and interacted with miR-26a. In ESCC tissues, lncRNA TPT1-AS1 level was obviously increased, while miR-26a level was decreased. Interestingly, lncRNA TPT1-AS1 level was not significantly correlated with miR-26a level but was positively correlated with HMGA1 mRNA, a target of miR-26a. In ESCC cell lines KYSE510 and KYSE-30, lncRNA TPT1-AS1 overexpression enhanced HMGA1 expression, while it had no effect on miR-26a expression. Cell migration and proliferation assays indicated that lncRNA TPT1-AS1 and HMGA1 overexpression promoted ESCC cell migration and invasion, while their effects were alleviated by miR-26a overexpression. The migration and invasion of ESCC cells were suppressed by lncRNA TPT1-AS1 knockdown. In conclusion, lncRNA TPT1-AS1 plays an oncogenic role in ESCC and might function by upregulating HMGA1 via sponging miR-26a.

## 1 Introduction

Esophageal cancer has been estimated to affect 572,034 new cases and has caused 508,585 deaths worldwide in 2018 [1]. Esophageal squamous-cell carcinoma (ESCC) is the common type of esophageal cancer [2]. Smoking, heavy alcohol consumption, and human papillomavirus infection are the main risk factors of ESCC [3,4]. Despite the continuous improvement in the medical standards in recent years, the 5 year survival rate of patients with esophageal cancer is not ideal [5,6,7]. Therefore, it is still necessary to develop novel diagnostic markers and therapeutic strategies.

It has been well established that the development of ESCC involves multiple molecular pathways [8]. The functional molecules involved in these pathways may be potential diagnostic markers and therapeutic targets [9,10]. Long (>200 nt) non-coding RNAs (lncRNAs) can function as gene regulators by interacting with DNA (e.g., promoters), RNA, or proteins [11]. Recently, mounting lncRNAs have been recognized as important regulators in ESCC. Accumulating evidence has proposed that lncRNAs play significant roles in the biological development of ESCC by regulating cell proliferation and apoptosis [12,13]. For instance, lncRNA small nucleolar host gene 1 (SNHG1), SNHG6, and SNHG16 have been reported to promote ESCC cell proliferation [14,15]. Cox univariate and multivariate analyses have revealed that SNHG1 is an independent prognostic factor for overall survival (OS) and disease-free survival in ESCC patients. The interaction between miRNA-21 and SNHG1 plays an important role in ESCC cell proliferation [16]. lncRNA SNHG1 promotes the development of cervical cancer cells. lncRNA SNHG1 knockdown decreases the proliferation, migration, and invasion of HeLa and C-33A cells [17]. lncRNA SNHG1 is upregulated in ESCC tissues and high SNHG1 expression is positively correlated with ESCC lymph node metastasis and decreased OS. CASC9 promotes ESCC growth by regulating the EZH2/CASC9 pathway [18], while KLF3-AS1 inhibits ESCC tumorigenesis by regulating the miR-185-5p/KLF3 axis [19]. TPT1-AS1, a lncRNA located on chromosome 13 with about 76,000 base pairs, has an oncogenic role in ovarian and cervical cancers [20,21,22]. Moreover, TPT1-AS1 accelerates the progression of colorectal cancer (CRC) by upregulating TPT1 levels and activating the FAK and JAK-STAT3 signaling pathways [23]. Tiang et al. has studied TPT1-AS1 and reported that TPT1-AS1 silencing suppressed gastric cancer development [22]. However, the effect of TPT1-AS1 on ESCC remains largely unknown. TPT1-AS1 is predicted to interact with miR-26a by IntaRNA software. Existing studies have revealed that miR-26a and its target HMGA1 are involved in the pathological progress of many cancers [24,25,26]. For instance, the interaction between miR-26a and its target gene HMGA1 might contribute to the malignant progression of human urothelial bladder cancer [27]. In addition, miR-26a-5p overexpression could be a novel therapy to improve coronary microembolization-induced myocardial damage. Studies have shown that HMGA1 is a target gene of miR-26a-5p. However, whether the TPT1-AS1/miR-26a axis plays a role in ESCC remains unclear. Here we attempted to verify the relationship between TPT1-AS1 and miR-26a and investigate the potential roles of TPT1-AS1 and miR-26a/HMGA1 axis in ESCC.

## 2 Methods

### 2.1 Research subjects

60 ESCC patients from Shanxi Cancer Hospital (Shanxi, China) between January 2017 and January 2019 were involved. Patients who had a history of malignancies or ESCC treatment or had other clinical diseases were excluded. The correlation between TPT1-AS1/miR-26a/HMGA1 expressions and clinicopathological factors of ESCC patients are listed in Tables 1–3.

**Table 1:** Association between TPT1-AS1 expression and clinicopathological features of ESCC patients

Characteristics	Total number (*n* = 60)	TPT1-AS1 expression		*P* value
		Low (*n* = 30)	High (*n* = 30)	
Age (years)				0.606
<55	30	16	14	
≥55	30	14	16	
Gender				0.438
Male	29	13	16	
Female	31	17	14	
Lymph node metastasis				0.114
Yes	24	9	15	
No	36	21	15	
Clinical stage				0.001
I–II	33	10	23	
III–IV	27	20	7	

The screening criteria were *P* < 0.05 and a fold change >1.

The study included 60 patients (29 males and 31 females). Among them, 30 patients were assigned in the high TPT1-AS1 expression group. Of these 30 patients, 15 patients had lymph node metastasis and 15 had no lymph node metastasis.

**Table 2:** Association between miR-26a expression and clinicopathological features of ESCC patients

Characteristics	Total number (*n* = 60)	miR-26a expression		*P* value
		Low (*n* = 30)	High (*n* = 30)	
Age (years)				0.121
<55	30	18	12	
≥55	30	12	18	
Gender				0.796
Male	29	14	15	
Female	31	16	15	
Lymph node metastasis				0.035
Yes	24	16	8	
No	36	14	22	
Clinical stage				0.020
I–II	33	21	12	
III–IV	27	9	18	

**Table 3:** Association between HMGA1 expression and clinicopathological features of ESCC patients

Characteristics	Total number (n = 60)	HMGA1 expression		*P* value
		Low (*n* = 30)	High (*n* = 30)	
Age (years)				1
<55	30	15	15	
≥55	30	15	15	
Gender				0.796
Male	29	14	15	
Female	31	16	15	
Lymph node metastasis				0.598
Yes	24	11	13	
No	36	19	17	
Clinical stage				0.020
I–II	33	12	21	
III–IV	27	18	9	


**Ethics approval and consent to participate:** The present study was approved by the Ethics Committee of Shanxi Cancer Hospital. The research has been carried out in accordance with the World Medical Association Declaration of Helsinki. All patients and healthy volunteers provided written informed consent prior to their inclusion within the study.

### 2.2 RNA interaction prediction

The interaction between TPT1-AS1 and miR-26a was predicted using IntaRNA software (http://rna.informatik.uni-freiburg.de/IntaRNA/Input.jsp).

### 2.3 ESCC tissue samples and cells

Fine needle biopsies under the guidance of MRI were performed to collect ESCC tissues and their matched adjacent normal-appearing tissues. The tissue sections were stained by hematoxylin and eosin, and their histopathological features were examined by three professional pathologists. The ESCC cancer tissues were diagnosed according to the World Health Organization’s classification for esophageal cancer [28]. The matched adjacent normal-appearing tissues were diagnosed with the criteria that there were no detectable cancer cells in the samples.

KYSE510 and KYSE-30 ESCC cell lines were from BFB Biotechnology Co., Ltd (China). They were cultured in RPMI-1640 medium with 10% FBS under 5% CO_2_ at 37°C. The cells were harvested from passages 3–6 for subsequent experiments. All experiments were performed with mycoplasma-free cells, which were authenticated by STR analysis.

### 2.4 Cell transfection

Short hairpin RNAs targeting TPT1-AS1 (shTPT1-AS1) and scrambled vector (shNC) from Invitrogen (Carlsbad, CA, USA) were used to knockdown TPT1-AS1. The expression vectors of TPT1-AS1 (NCBI accession: NR_024458.1) and HMGA1 (NCBI accession: KJ891364.1) were constructed by Sangon Biotech (China). The miR-26a mimic (5′-UUCAAGUAAUCCAGGAUAGGCU-3′) and negative control (5′-GUACGUAGCUAGUACGGUCCCA-3′) were designed by Invitrogen (USA). The HMGA1 overexpression plasmid was purchased from Gikai Gene Company (GV492, Shanghai, China) (Table 4). Lipofectamine 2000 (Invitrogen, Carlsbad, CA, USA) was used for cell transfection.

**Table 4:** Inserted sequence of HMGA1 overexpression plasmid

ATGAGTGAGTCGAGCTCG AAGTCCAGCCAGCCCT TGGCCTCC AAGCAG GAAAAGGACGGCACTGAGAAGC GGGGCCGGGGCAGG CCGCGCAAG CAGCCTCCGGTGAGTCCCGGGACAGCGCTGGTAGGGAG TCAGAAGGA GCCCAGC GAAGTGCCAACACCTAAGAGACCTCGG GGCCGACC AAAGG GAAGCAAAA ACAAGGGTGCTGCCAAGACCCG GAAAACCACCACA ACT CCAGGA AGGAAACCAAGGGGCAGACCC AAAAAACTGGAGAAGGAGG AA GAGGAGGGCATCTCGCAGGA GTCCTCGGAGGAGGAGCAG

### 2.5 Dual luciferase reporter assay

Dual luciferase reporter assay was performed following a previous work [29]. Briefly, pGL3 Promoter Luciferase Reporter Vector (Promega Corporation) was used to construct TPT1-AS1 vector. KYSE510 cells were transfected with pGL3-TPT1-AS1-promoter + pRL-TK + NC miRNA (NC group) or pGL3-TPT1-AS1-promoter + pRL-TK + miR-26a mimic (miR-26a group) using Lipofectamine 2000. The luciferase activity was determined using LucPair™ Duo-Luciferase Assay Kit (GeneCopoeia), and the firefly/Renilla activity ratio was calculated.

HMGA1 wild-type and mutant-type luciferase reporter vector targeting the miR-26a binding site were constructed. The vectors and miR-26a mimics were co-transfected into cells using Lipofectamine 2000 reagent, and luciferase activities were detected 48 h later using the dual luciferase reporter system (Promega, USA).

### 2.6 RNA preparations and RT-qPCR

Total RNAs and miRNAs were extracted from KYSE510 cells and tissue samples using Direct-zol RNA Kit (R2061, Zymo Research) and PureLink miRNA Isolation Kit (K157001, Thermo Fisher Scientific), respectively. The cDNA samples were obtained by using QuantiTect Reverse Transcription Kit (205311, QIAGEN). RT-qPCR reactions were performed with KAPA SYBR® FAST qPCR Master Mix (2X) Kit (KR0390, Kapa Biosystems) or All-in-One™ miRNA qRT-PCR Detection Kit (QP015/AOMD-Q020, GeneCopoeia). GAPDH and U6 were selected as the internal controls for regular genes and miRNA, respectively. The corresponding primer sequences were 5′-CGTTTGGACCCCTGTCTTGGAC-3′ (forward) and 5′-CAGAAAGAAAGCAGGTCATT-3′ (reverse) for TPT1-AS1; 5′-TGAGTCGAGCTCGAAGTCCAG-3′ (forward) and 5′-CTTAGGTGTTGGCACTTCGC-3′ (reverse) for HMGA1; and 5′-GTCTCCTCTGACTTCAACAGC-3′ (forward) and 5′-CCACCCTGTTGCTGTAGCCAA-3′ (reverse) for GAPDH. The forward miR-26a primer was 5′-UUCAAGUAAUCCAGGAUAG-3′. The reverse miR-26a primer and U6 primers were from the kit. The PCR reaction conditions were 1 min at 95°C followed by 40 cycles of 95°C for 10 s and 58°C for 50 s. Each experiment was performed with 3 replicates, and the relative expression level was calculated using the 2^−ΔΔCt^ method.

### 2.7 Subcellular fractionation

The nuclear and cytoplasm extracts of KYSE510 cells were isolated using NE-PER Nuclear and Cytoplasmic Extraction Reagents kit (pierce-78835, Pierce, USA). The TPT1-AS1, U6, and GAPDH levels in the nuclear and cytoplasmic extracts were detected by RT-qPCR.

### 2.8 Western blotting

Total proteins were isolated from *in vitro* cultivated cells and quantified using bicinchoninic acid assay method. 50 μg (10 μL) of total proteins were loaded to each sample well and separated by 10% SDS-PAGE. The separated proteins were transferred onto PVDF membranes, which were blocked with 5% nonfat dry milk for 2 h and incubated with antibodies against GAPDH (ab9845, Abcam) and HMGA1 (ab226850, Abcam) at 4°C for 18 h, followed by incubation with secondary antibody (ab6721, Abcam) at room temperature for 2 h. The target protein signals were detected using the enhanced chemiluminescence system (Amersham).

### 2.9 Transwell assays

Transwell assays were performed using KYSE510 and KYSE-30 cells collected at 48 h post-transfection using Transwell inserts (8 µm, 3415, Corning). For invasion assay, the Transwell inserts were pre-treated with 50 μL/well Matrigel (dilution rate = 1:3) at 37°C for 6 h. For migration assay, the uncoated Transwell inserts were used. 4,000 cells were seeded into the upper chamber, and RPMI-1640 media with 20% FBS were added into the lower chamber. 12 h later, the lower surface of membranes was stained using crystal violet (0.1%, Sigma-Aldrich) for 20 min. Images were analyzed using ImageJ software to measure the percentage of the area of cells present in each insert.

### 2.10 Statistics

The experimental data (three replicates) were analyzed using SPSS 17.0 software. Data from ESCC tissue samples were analyzed using paired *t* test and Pearson’s correlation coefficient. One-tailed *t*-test was used in the study. Unpaired *t* test was applied to compare the difference between two groups. ANOVA was used to compare the difference among multiple groups. The threshold of *p*-value was set to 0.05.

## 3 Results

### 3.1 TPT1-AS1 interacted with miR-26a

**Figure 1:**
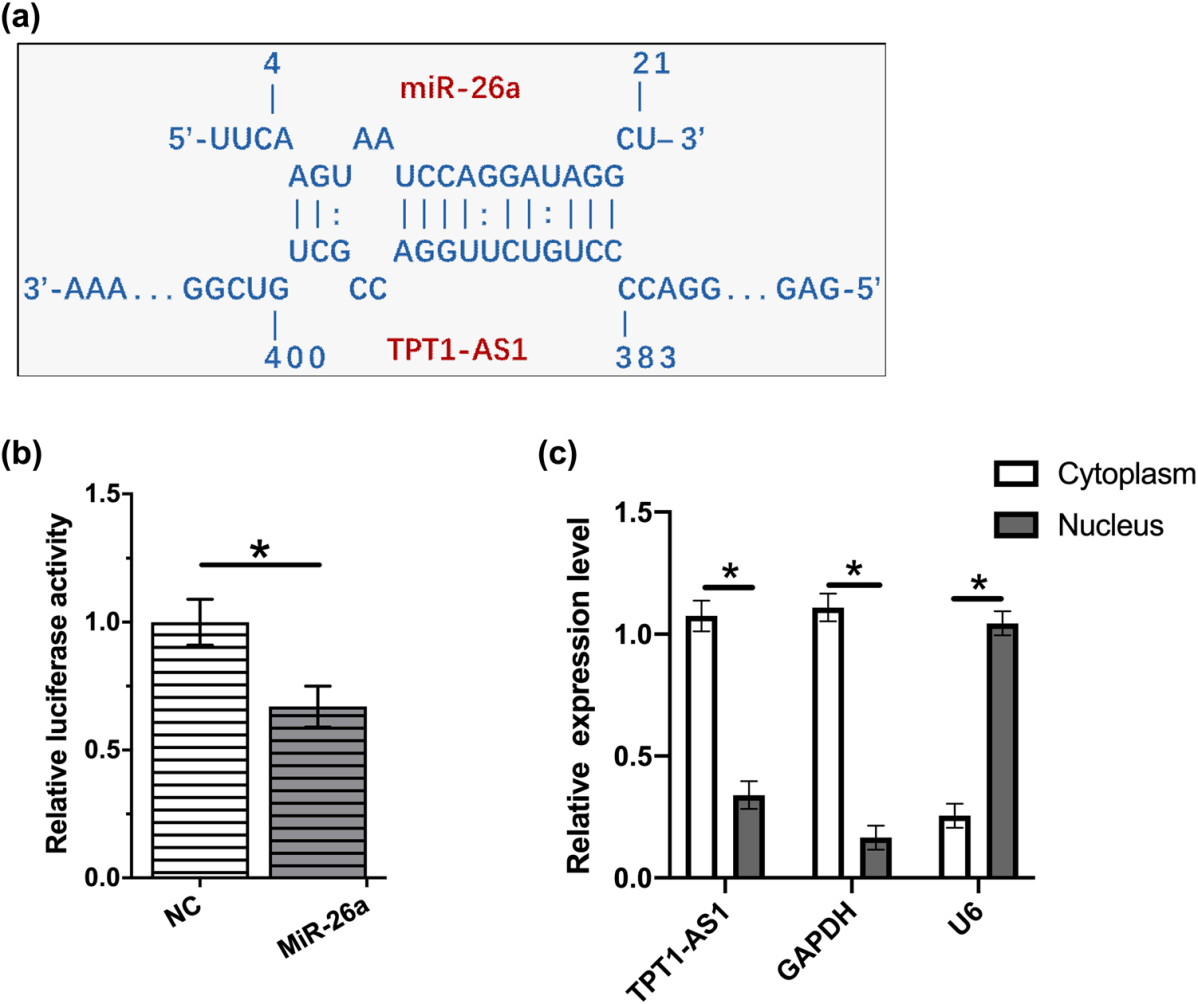
TPT1-AS1 interacted with miR-26a. (a) TPT1-AS1 was predicted to interact with miR-26a. (b) KYSE510 cells were transfected with pGL3-TPT1-AS1-promoter + pRL-TK + NC miRNA or pGL3-TPT1-AS1-promoter + pRL-TK + miR-26a mimic. Luciferase activity was assessed by using the corresponding assay kit. (c) TPT1-AS1 level in the nucleus and cytoplasm of ESCC cells. *, *p* < 0.05.

**Figure A1:**
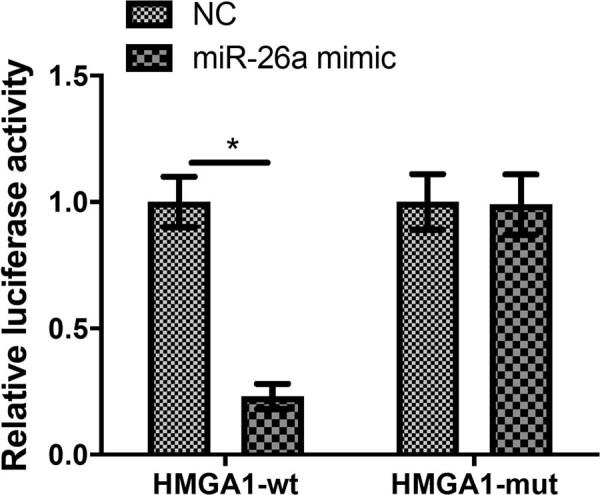
Luciferase assays in ESCC cells co-transfected with wild-type or mutant HMGA1 and miR-26a or NC. MiR-26a overexpression significantly reduced the luciferase activity of the HMGA1-wt vector.

TPT1-AS1 was predicted to interact with miR-26a by IntaRNA (Figure 1a). As shown in Figure 1b, miR-26a overexpression markedly inhibited the luciferase activity in KYSE510 cells transfected with pGL3-TPT1-AS1-promoter and pRL-TK, indicating that TPT1-AS1 interacted with miR-26a. Moreover, we isolated the nuclear and cytoplasmic extracts from ESCC cell line KYSE510. Subsequent RT-qPCR assay indicated that TPT1-AS1 was mainly localized in the cytoplasm of KYSE510 cells (Figure 1c). MiR-26a overexpression significantly reduced the luciferase activity of the HMGA1-wt vector but failed to decrease that of the HMGA1-mut (Figure A1).

### 3.2 TPT1-AS1 level was increased while miR-26a level was reduced in ESCC tissues

**Figure 2:**
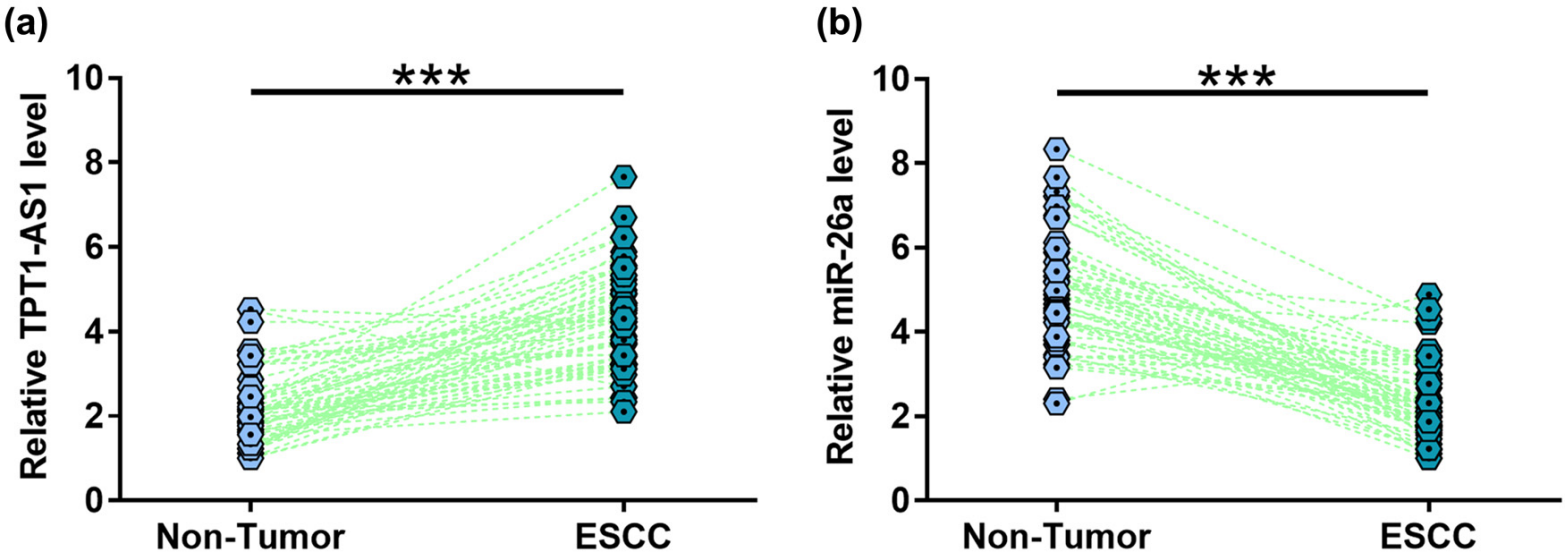
TPT1-AS1 level was increased, while miR-26a level was reduced in ESCC tissues. TPT1-AS1 (a) and miR-26a (b) levels in ESCC and matched non-tumor tissues were measured by RT-qPCR. ***, *p* < 0.001.

**Figure A3:**
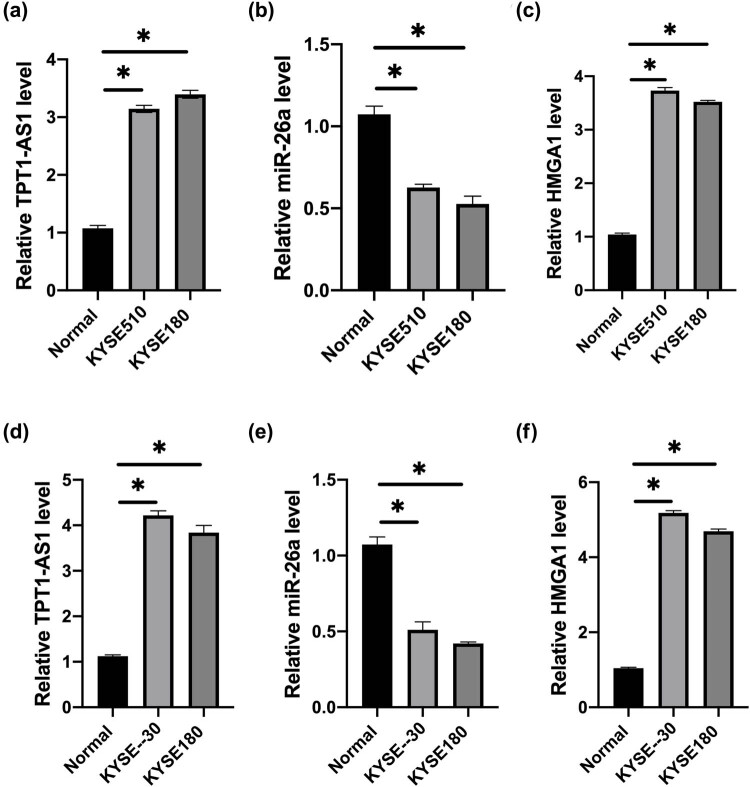
The expression levels of TPT1-AS1 and miR-26a/HMGA1 in these two cells were compared with other ESCC cells lines and normal cell line. TPT1-AS1 (A, D), miR-26a (B, E) and HMGA (C, F) levels in ESCC cells and normal cell line were determined by RT-qPCR.

TPT1-AS1 and miR-26a levels in ESCC and matched non-tumor tissues were assessed. The results indicated that TPT1-AS1 level was markedly increased in ESCC tissues (Figure 2a), while miR-26a level was apparently reduced in ESCC tissues (Figure 2b). The expression levels of TPT1-AS1 and miR-26a/HMGA1 in these two cells were compared with KYSE180 cells and normal cells. The results indicated that TPT1-AS1 level was increased in KYSE180 cells and KYSE510 cells (Figure A3a), while miR-26a level was reduced in KYSE180 cells and KYSE510 cells (Figure A3b). In addition, HMGA level was increased in KYSE180 cells and KYSE510 cells (Figure A3c). Moreover, TPT1-AS1 level was increased in KYSE180 cells and KYSE-30 cells (Figure A3d), while miR-26a level was reduced in KYSE180 cells and KYSE-30 cells (Figure A3e). Moreover, HMGA level was increased in KYSE180 cells and KYSE-30 cells (Figure A3f).

### 3.3 TPT1-AS1 enhanced HMGA1 expression in ESCC cells by sponging miR-26a

**Figure 3:**
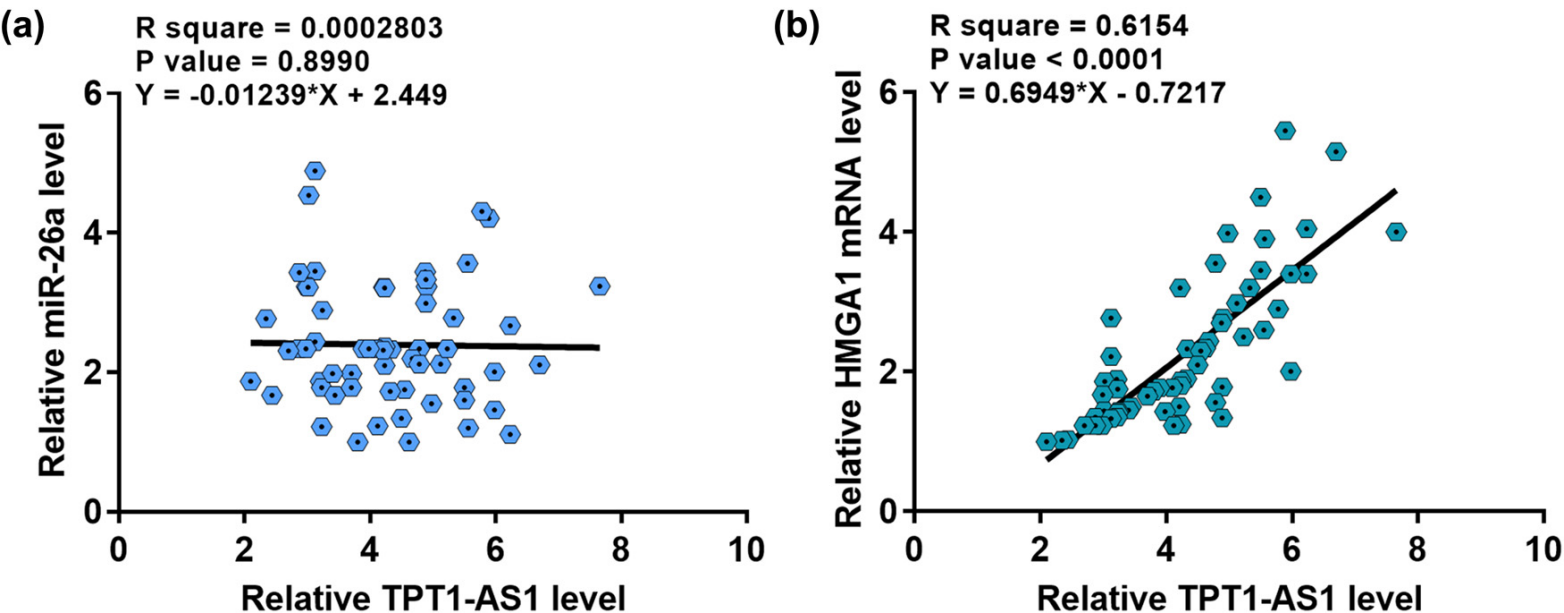
TPT1-AS1 was positively correlated with HMGA1 mRNA. (a) The correlation between TPT1-AS1 and miR-26a across ESCC tissues. (b) The correlation between TPT1-AS1 and HMGA1 mRNA across ESCC tissues.

**Figure 4:**
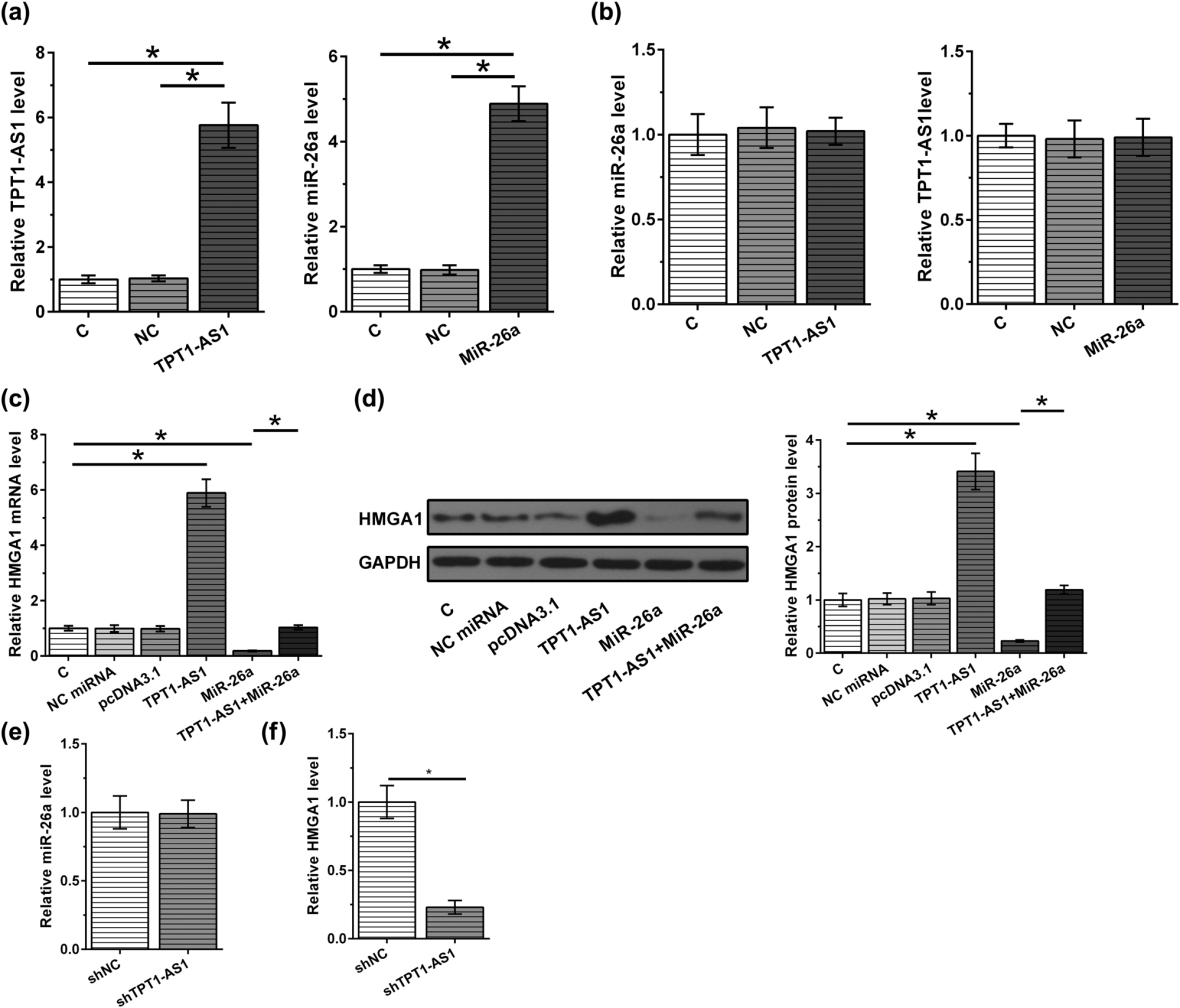
TPT1-AS1 enhanced HMGA1 expression by sponging miR-26a. TPT1-AS1 expression vector and miR-26a mimic were transfected into KYSE510 cells. (a and b) TPT1-AS1 and miR-26a levels were assessed by RT-qPCR. (c and d) HMGA1 expression level was assessed by RT-qPCR and western blotting. (e and f) The expressions of miR-26a and HMGA1 were assessed by RT-qPCR in KYSE510 cells transacted with shTPT1-AS1. *, *p* < 0.05.

The correlation among TPT1-AS1, miR-26a, and HMGA1 levels were assessed. As shown in Figure 3a, TPT1-AS1 level in ESCC tissues was not obviously correlated with miR-26a level. Interestingly, TPT1-AS1 level in ESCC tissues was positively correlated with HMGA1 mRNA, a target of miR-26a (Figure 3b). We then transfected KYSE510 cells with TPT1-AS1 expression vector or miR-26a mimic. As shown in Figure 4a, TPT1-AS1 overexpression and miR-26a mimic remarkably upregulated TPT1-AS1 and miR-26a levels in KYSE510 cells, respectively. As shown in Figure 4b, TPT1-AS1 and miR-26a overexpression did not affect the expression levels of each other. In addition, we also assessed the effects of TPT1-AS1 and miR-26a overexpression on HMGA1. The results indicated that TPT1-AS1 overexpression promoted HMGA1 mRNA and protein levels, while miR-26a overexpression inhibited HMGA1 mRNA and protein levels (Figure 4c and d). Moreover, miR-26a overexpression attenuated the effects of TPT1-AS1 overexpression on HMGA1 mRNA and protein expression (Figure 4c and d). Furthermore, TPT1-AS1 knockdown had no effect on miR-26a expression (Figure 4e) but decreased HMGA1 expression (Figure 4f). Collectively, TPT1-AS1 might enhance HMGA1 expression by sponging miR-26a.

### 3.4 TPT1-AS1 enhanced ESCC cell migration and invasion by regulating the miR-26a/HMGA1 axis

**Figure 5:**
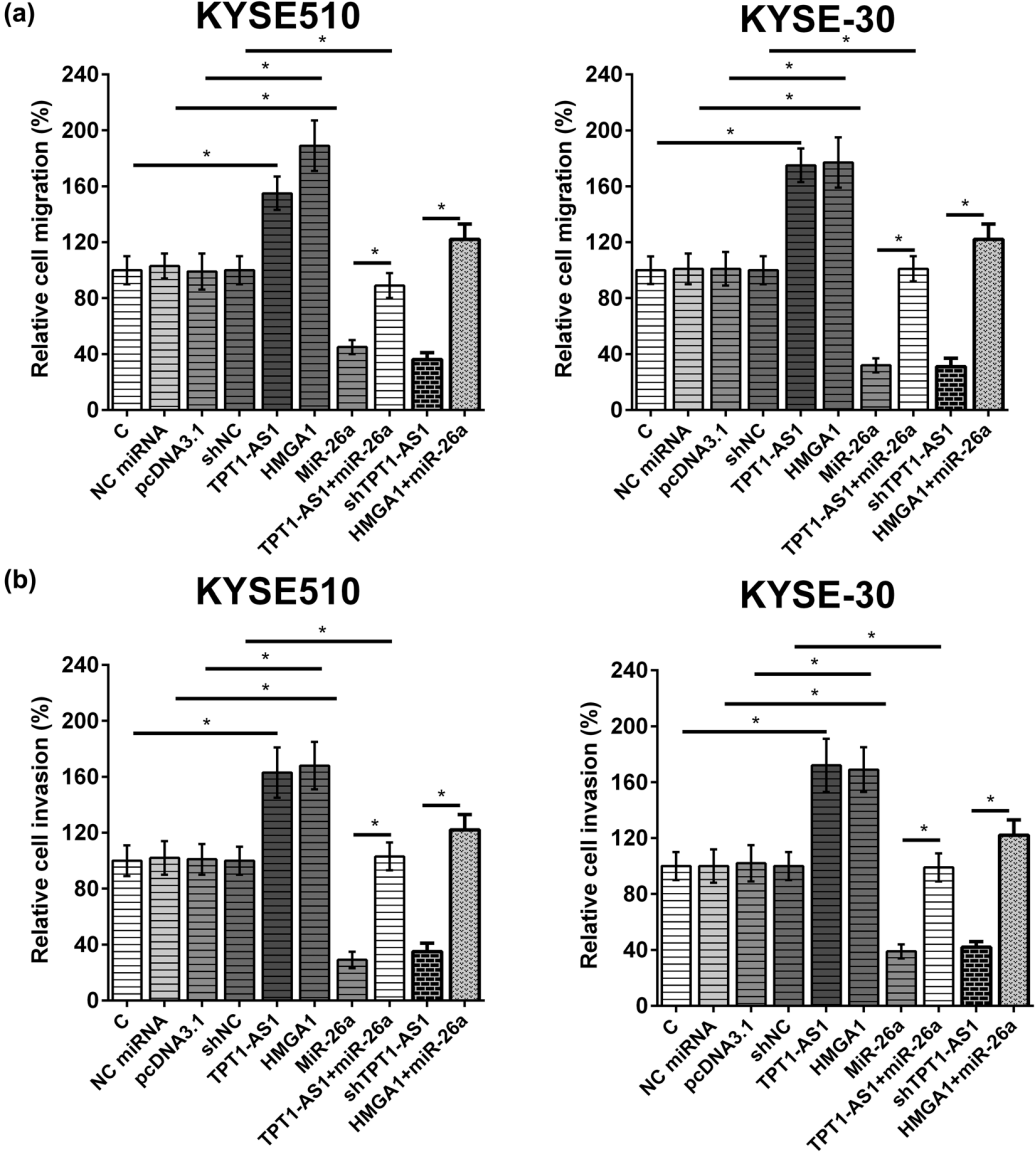
TPT1-AS1 regulated the miR-26a/HMGA1 axis to enhance KYSE510 and KYSE-30 cell migration and invasion. ESCC cells migration (a) and invasion (b) abilities were determined by Transwell assays. *, *p* < 0.05.

The function of TPT1-AS1 in ESCC cells was explored. TPT1-AS1 and HMGA1 overexpression markedly promoted migration and invasion of ESCC cells, while miR-26a overexpression and TPT1-AS1 knockdown obviously suppressed migration and invasion of ESCC cells. Moreover, the effects of TPT1-AS1 and HMGA1 overexpression on migration and invasion were alleviated by miR-26a overexpression (Figure 5a and b).

## 4 Discussion

Our study mainly explored the role of TPT1-AS1 in ESCC. We revealed that TPT1-AS1 is remarkably increased in ESCC, and it could enhance ESCC cell migration and invasion by regulating the miR-26a/HMGA1 axis. Therefore, TPT1-AS1 might be a possible therapeutic target for ESCC.

Previous studies have shown that TPT1-AS1 knockdown significantly inhibits proliferation and cell cycle G1/S transition of SGC-7901 and MGC-803 cells. TPT1-AS1 diminishes cell proliferation and sensitizes cells to chemotherapy by sponging miR-3156-5p and upregulating CASP2. In addition, it has been reported that TPT1-AS1 induces epithelial ovarian cancer (EOC) tumor growth and metastasis through TPT1 and downstream PI3K/AKT signaling and that TPT1-AS1 might be a promising therapeutic target for EOC. Recent studies have explored the involvement of TPT1-AS1 in ovarian and cervical cancers [20,21]. In ovarian cancer, TPT1-AS1 is upregulated and induces TPT1 expression to promote cancer cell metastasis [21]. In cervical cancer, TPT1-AS1 is increased and sponges miR-324-5p [30] to promote tumor metastasis and growth [20]. Previous studies have also shown that TPT1-AS1 enhances EOC cell proliferation, migration, and invasion via the TPT1/PI3K/AKT signaling pathway *in vitro*. In addition, TPT1-AS1 promotes tumor progression and metastasis in CRC by upregulating TPT1 level and activating the FAK and JAK-STAT3 signaling pathways. The phenotypic changes in pathways related to migration and invasion remain to be further elucidated [22,31]. Here we observed that TPT1-AS1 is remarkably increased in ESCC tissues, and its overexpression enhances ESCC cell migration and invasion. Therefore, TPT1-AS1 also has an oncogenic function in ESCC. Moreover, we uncovered that TPT1-AS1 interacts with miR-26a. Existing studies have found that the role of miR-26a in cancers is differentiated. In CRC, miR-26a is apparently downregulated and can regulate FUT4 expression to suppress cancer cell aggressiveness [32]. In triple-negative breast cancer, miR-26a is downregulated and can target metadherin to suppress cancer cell migration and proliferation [33]. However, miR-26a is increased in ovarian cancer and promotes cancer cell proliferation [34]. Here we observed that miR-26a is decreased in ESCC tissues, and its overexpression represses ESCC cell migration and invasion, consistent with a recent report [35].

Studies have found that miR-26a suppresses bladder cancer by regulating its target HMGA1 [26]. HMGA1 can regulate various genes due to its ability to alter chromatin structures. HMGA1 overexpression is a hallmark of human cancers and exhibits a pivotal role in cell transformation [36]. miR-26a downregulates HMGA1 by targeting its 3′-UTR, and HMGA1 knockdown significantly suppresses the migration and invasion of two osteosarcoma cell lines *in vitro* [37]. Moreover, we uncovered that TPT1-AS1 might play its oncogenic function by modulating the miR-26a/HMGA1 axis. Further studies are needed to explore other potential mechanisms.

Our study also has some limitations. First, our sample size of ESCC patients is small, and all patients are Han Chinese. Therefore, a bigger sample size with different ethnic backgrounds is needed to further confirm our conclusion. In addition, *in vivo* animal experiments are needed to assess the role of TPT1-AS1 in tumor metastasis. The effect of HMGA1 knockdown on ESCC cells migration and invasion should be confirmed in the future.

## 5 Conclusion

TPT1-AS1 level is markedly increased in ESCC tissues, and its overexpression enhances ESCC cell migration and invasion via modulating the miR-26a/HMGA1 axis.

## List of abbreviations

CR: Ccolorectal cancer
EOC: epithelial ovarian cancer
ESCC: esophageal squamous-cell carcinoma
lncRNAs: long (>200nt) non-coding RNAs
NC: negative control
OS: Overall Survival
SNHG1: small nucleolar host gene 1

## Figures and Tables

**Figure A2:**
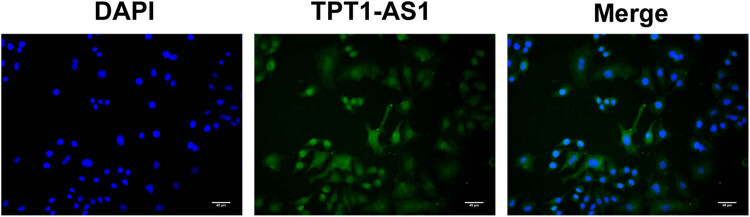
TPT1-AS1 localization in KYSE510 cells. RNA-FISH revealed that TPT1-AS1 is mainly colocalized in the cytoplasm of KYSE510 cells.
